# The microbiome of the pregnant uterus in Holstein dairy heifers and cows assessed by bacterial culture and 16S ribosomal RNA gene sequencing

**DOI:** 10.3389/fmicb.2024.1385497

**Published:** 2024-05-15

**Authors:** Joao G. N. Moraes, Tamara Gull, Aaron C. Ericsson, Scott E. Poock, Monica O. Caldeira, Matthew C. Lucy

**Affiliations:** ^1^Department of Animal and Food Sciences, Oklahoma State University, Stillwater, OK, United States; ^2^College of Veterinary Medicine, University of Missouri, Columbia, MO, United States; ^3^Division of Animal Sciences, University of Missouri, Columbia, MO, United States

**Keywords:** microbiome, pregnancy, uterus, cow, heifer

## Abstract

**Introduction:**

The possibility that there is a resident and stable commensal microbiome within the pregnant uterus has been supported and refuted by a series of recent studies. One element of most of the initial studies was that they were based primarily on 16S rRNA gene sequencing from bacteria. To account for this limitation, the current study performed both bacterial culture and 16S rRNA gene sequencing in a side-by-side manner (e.g., same tissues isolated from the same animal).

**Methods:**

The uteruses of 10 mid-pregnant (156 ± 5 d of gestation) Holstein heifers and cows were collected following slaughter. The external surface of the reproductive tract (positive control for contamination during tissue collection) as well as tissues within the pregnant uterus (placentome, inter-cotyledonary placenta, inter-caruncular endometrium, amnionic fluid, allantoic fluid, fetal abomasum content, and fetal meconium) were sampled for bacterial culture and 16S rRNA gene sequencing.

**Results:**

There were 87 unique bacterial species cultured from the external surface of the pregnant reproductive tract (contamination control) and 12 bacterial species cultured from pregnancy tissues. Six out of 10 cattle (60%) exhibited bacterial growth in at least one location within the pregnant uterus. For the metataxonomic results (16S rRNA gene sequencing), a low targeted microbial biomass was identified. Analyses of the detected amplicon sequence variants (ASV) revealed that there were: (1) genera that were prevalent on both the external surface and within the pregnant uterus; (2) genera that were prevalent on the external surface but either not detected or had very low prevalence within the pregnant uterus; and (3) genera that were either not detected or had low prevalence on the external surface but found with relatively high prevalence within the pregnant uterus.

**Conclusion:**

There are a small number of viable bacteria in the pregnant uterus. The 16S rRNA gene sequencing detected a microbial community within the pregnant uterus but with a low biomass. These results are consistent with recent studies of the pregnant bovine uterus and leave open the question of whether there is adequate microbial mass to significantly affect the biology of the normal healthy bovine pregnancy.

## 1 Introduction

The microbiome consists of pathogenic and commensal microorganisms (bacteria, fungi, protozoa, and viruses) that occupy specific ecological niches throughout the body (Fischbach, 2018). Under nonpathogenic circumstances, the commensal microbiome may serve an important functional role as it does in the rumen and intestine where microorganisms are involved in digestion (Pitta et al., 2018). There is also the potential for disease if the normal commensal relationship between host and microbiome is disturbed and there is overgrowth of specific organisms (Plaizier et al., 2018). Although the presence of the microbiome has been recognized for years, it was not until recently that the importance of the microbiome to the physiology of the organism was fully recognized (Sonnenburg and Bäckhed, 2016). The most widely studied microbiota include those found in the intestine, oral cavity, respiratory tract, skin, and vagina.

Although the importance of the vaginal microbiome had been well established (Ma et al., 2012), the uterus has traditionally been regarded as a sterile environment (Escherich, 1988), implying that the cervix must function as an effective barrier, allowing sperm to pass into the uterus but limiting the ascent of bacteria from the vagina into the uterus (Lacroix et al., 2020). Organisms that entered the uterus were believed to be efficiently killed by resident immune cells to create a sterile uterine lumen (Mei et al., 2019). The traditional belief in a “sterile womb,” initially proposed based on culture-depended methods, has been called into question by advances in next-generation sequencing technologies, which have enabled the detection of bacterial DNA within the uterine lumen and pregnancy tissues (Perez-Muñoz et al., 2017). This raises the intriguing possibility that a uterine microbiome or the presence of bacterial DNA could influence normal embryonic development and pregnancy outcomes. Nonetheless, compelling evidence supporting the existence of a uterine microbiome with significant functional implications in uterine biology and pregnancy development remains limited in the current literature. This limitation stems from challenges related to “low biomass” experiments, inadequate control measures for contamination, and failure to demonstrate bacterial viability (Perez-Muñoz et al., 2017).

To achieve a successful pregnancy, the developing embryo must implant into the uterine endometrium, and therefore the composition of the endometrial microbiota has been hypothesized to influence pregnancy outcomes. Indeed, several studies have demonstrated an association between infertility and uterine microbiome in humans (reviewed by Benner et al., 2018). In cattle, postpartum uterine infection significantly reduces conception rates and increases rates of embryonic mortality (Galvão et al., 2019; Sheldon et al., 2020; Moraes et al., 2021). Furthermore, specific viruses, bacteria, and fungi can cause abortion (Givens and Marley, 2008). Hence, the notion of a resident and stable commensal microbiome within the uterus influencing reproductive efficiency has intrigued researchers. The publication of a paper by Aagaard et al. (2014) that demonstrated a placental microbiome of human pregnancy created intense interest and opened the field to further investigation. We published some of the first work in the cow to show evidence of bacterial DNA within the pregnant uterus (Moore et al., 2017). More recent studies concluded that there is a microbiome within the gastrointestinal tract and amnionic fluid of the bovine fetus (Guzman et al., 2020; Husso et al., 2021). In addition to these publications in human and bovine, there have been numerous additional studies in which investigators either supported or refuted the notion that there is a microbiome of pregnancy (Baker et al., 2018; Silverstein and Mysorekar, 2021; Walter and Hornef, 2021).

One element of most of these studies, including ours, was that they were based primarily on 16S rRNA gene sequences from bacteria. A common criticism was that (1) the presence of bacterial DNA does not necessarily equate to living organisms and; (2) the microbial community within the healthy uterus has a very low biomass that is near the threshold found in negative controls (Eisenhofer et al., 2019; O’Callaghan et al., 2020; Lietaer et al., 2021). The objective of the current study was to address the first question by performing bacterial culture and 16S rRNA gene sequencing in a side-by-side manner (same tissues isolated from the same animal). Bacterial culture has a limited range for detection of organisms relative to 16S rRNA gene sequencing but we nonetheless hypothesized that the two techniques would have some overlap with respect to results (similar organisms would be detected across both methods).

## 2 Materials and methods

### 2.1 Study design

Study procedures were approved by the University of Missouri Institutional Animal Care and Use Committee (Protocol number 9635). A total of 10 mid-pregnant (151 to 161 d) Holstein heifers (*n* = 5) and cows (*n* = 5) were enrolled in the study. The cattle were humanely slaughtered by captive bolt stunning and exsanguination. The uterus was removed from the abdomen by cutting it from the broad ligament and transecting the vagina. The entire tract was wrapped in surgical drape, placed on ice, and brought to a pathology laboratory immediately upon collection. The external surface (EXT) of the uterus was sampled for bacteriological culture by using a sterile culture swab. Afterwards, a tissue sample from the uterine surface near the tip of the horn was collected using sterile forceps and scalpel, inserted into a sterile cryovial and frozen in liquid nitrogen to be used for 16S rRNA gene sequencing. The purpose of collecting external (EXT) samples for bacteriology and 16S rRNA gene sequencing was to assess environmental contamination levels during animal slaughter and reproductive tract collection.

For the collection of pregnancy-associated samples, the outside of the uterus was cleaned and disinfected with povidone-iodine, and samples of amnionic fluid (AM_F; site near the head of the fetus) and allantoic fluid (AL_F; site near the tip of the uterine horn) were taken by aspirating with a sterile single-use hypodermic needle and syringe. The amniotic fluid directly surrounds the fetus, and the allantoic fluid is primarily a result of embryonic metabolism, mostly derived from plasma filtrated by the kidneys. Thus, studying their microbiome can provide information about the fetal microbial environment.

Tissue samples from placentome (PLT), inter-cotyledonary placenta (IC_P), and inter-caruncular endometrium (IC_E) were collected to evaluate the microbial load of important structures mediating maternal-fetal interactions. Tissue samples of the fetal abomasum (ABO) and swab samples of fetal meconium (MEC) were also collected to evaluate the possible microbial load of the fetal digestive system. In every case, samples were processed immediately for bacteriological culture and a second sample was placed into a cryovial, frozen in liquid nitrogen, stored at −80°C and used for 16S rRNA gene sequencing.

### 2.2 Bacteriology

Tissue specimens were sterilely ground in thioglycolate broth in a single-use disposable tissue grinder. Sterile swabs were used to inoculate the various agars and 1 mL of the tissue slurry was inoculated into a fresh 9 mL tube of thioglycolate broth for incubation. Swab specimens were used to directly inoculate the various agars and, following that, the swabs were placed in thioglycolate broth for incubation. All samples were plated onto tryptic soy agar with 5% sheep blood (TSA), MacConkey agar, phenylethyl alcohol agar (PEA), and thioglycolate broth for incubation under aerobic conditions. Aerobic cultures were incubated at 36°C in a standard ambient air incubator. Capnophilic cultures were maintained at 36°C under 5% CO_2_. *Campylobacter* cultures were placed in Mitsubishi boxes equipped with a microaerophilic sachet (Mitsubishi AnaeroPak MicroAero gas generator, Remel, Lenexa, KS), providing 6–12% O_2_ and 5–8% CO_2_, and then incubated at 42°C for enteric *Campylobacter* and 35°C for reproductive *Campylobacter*. Samples were also plated onto TSA and PEA for incubation under anaerobic conditions; chocolate agar, Hayflick agar, and BHI broth for incubation under 5% CO2; and selective *Campylobacter* agar for incubation under microaerophilic conditions. Anaerobic cultures were held in Mitsubishi boxes using a Mitsubishi AnaeroPack anaerobic gas generator (Remel), and anaerobic conditions (<1% O_2_, >15% CO_2_) were verified using anaerobic indicators (Remel). All bacterial culture media used were sourced from Remel, except for the reproductive *Campylobacter* medium, obtained pre-reduced from Anaerobe Systems (Morgan Hill, CA). The various agars and broth allowed the isolation of the greatest variety of organisms from the samples, since this study was intended, in part, to compare conventional culture-based methods of detection with molecular methods. Media were incubated for 7 days and evaluated daily. All isolates were identified via MALDI-TOF mass spectrometry, standard biochemical tests, and/or 16S rRNA sequencing.

### 2.3 16S rRNA gene sequencing and analysis

A manual precipitation protocol was used for DNA extraction (Yu and Morrison, 2004). Library construction and sequencing were performed by the University of Missouri DNA Core. A Qubit dsDNA BR Assay (Life Technologies, Carlsbad, CA) was used to determine DNA concentration. Samples were normalized to 3.51 ng/μL DNA for PCR amplification. The V4 hypervariable region of the 16S rRNA gene was amplified using single-indexed universal primers [U515F (GTGCCAGCMGCCGCGGTAA); 806R (GGACTACHVGGGTWTCTAAT)] with standard adapter sequences (Illumina Inc., San Diego, CA). The PCR program for amplification was: 98°C (3:00) + [98°C (0:15) + 50°C (0:30) + 72°C (0:30)] × 40 cycles + 72°C (7:00; min:s). The V4 region of the 16S rRNA gene was selected for library generation because this region yields optimal community clustering (Caporaso et al., 2011). The Illumina MiSeq platform (V2 chemistry with 2 × 250-bp paired-end reads) was used to sequence pooled amplicons. Amplicon sequences of the V4 hypervariable region of the 16S rRNA gene were processed and analyzed using QIIME2 (version 2020.6^1^) (Bolyen et al., 2019). Fastq files containing forward and reverse sequences were imported into QIIME2 and demultiplexed to assign sequences to samples. The plugin cutadapt (Martin, 2011) was used to trim off PCR primers (515F/806R) from raw sequences and to remove reads in which no adapter was found. QIIME2 Divisive Amplicon Denoising Algorithm (DADA2) plugin was used for detecting and correcting Illumina amplicon sequencing errors (Callahan et al., 2016). QIIME2 quality-control plugin was used to exclude contaminant sequences such as host sequences (e.g., cow DNA) and non-targeted (e.g., non-bacterial) sequences. Greengenes^2^ operational taxonomic unit (OTU) reference sequences (99% sequence identity) were used for quality control. Sequences filtered-out during this step were investigated using the NCBI BLAST nucleotide database^3^ to ensure that only contaminant sequences were removed.

To perform phylogenetic diversity analyses, a rooted phylogenetic tree was generated using QIIME2 phylogeny function after samples were rarefied to 200 sequences per sample. This rarefaction strategy aimed to maximize the number of sequences retained per sample while minimizing the exclusion of samples. Pairwise comparisons for alpha diversity measures [Pielou’s Evenness (Pielou, 1966) and Faith’s Phylogenetic Diversity (Faith, 1992)] were computed using the Kruskal-Wallis test. The unweighted UniFrac distances, a measure of beta diversity (Lozupone and Knight, 2005; Lozupone et al., 2007) were also calculated, and PERMANOVA was used in pairwise comparisons to evaluate beta-diversity group distances. Furthermore, principal coordinate analysis (PCoA) plot for the unweighted UniFrac distance was generated using Emperor (Vázquez-Baeza et al., 2013, 2017) to aid on data visualization and interpretation.

A pre-formatted taxonomy classifier (Bokulich et al., 2020) was used for assigning taxonomy classification to the 16S rRNA amplicon sequences (Pruesse et al., 2007; Pedregosa et al., 2012; Quast et al., 2013; Bokulich et al., 2018), and an amplicon sequence variant (ASV) table was generated (McDonald et al., 2012). Amplicon sequence variants (ASVs) sharing the same taxa were collapsed together (at the species level) using the QIIME2 taxa collapse function.

Differential abundance analyses on the identified ASVs was performed using the Analysis of Composition of Microbes (ANCOM) statistical framework (Mandal et al., 2015). For ANCOM, data was pre-processed to remove features with low reads (less than 10 reads across all samples), rarely observed (present in less than 2 samples), and with low variance (less than 10e-4). Because ANCOM is based on log ratios, QIIME2 add-pseudocount plugin was used to add one count to every feature, allowing ANCOM analysis to be performed on features with zero counts. Pairwise comparisons with ANCOM were used to compare the microbiome of sample from different locations.

The identified ASV were collapsed to the species level (i.e., the reads from species sharing the same species were summed together) for further analyses that included the prevalence (number of individuals with a specific bacterial genus within a specific tissue). The ASV with low reads (less than 10 reads across all samples) and present in less than 2 individuals within a tissue sample were removed from this summary. The method described by Davis et al. (2018) was used to correct for background reagent contamination in the samples. The method is based on the observation that DNA amplification of 16S rRNA from contaminating species is expected to decrease in a log-linear manner with the total number of sequence reads. The genera detected within 10 or more individual samples across all tissues were tested using this method. The contaminating genera were removed from the summary of the prevalence for individual genera. Typical habitats for selected genera were based on available data from BacDive (The Bacterial Diversity Database^4^) and other web-based resources.

## 3 Results

### 3.1 Bacteriology

Bacteria were cultured from all EXT samples. There were 87 unique bacterial species cultured from at least one animal (Supplementary Table S1) and 23 species were cultured from more than one animal (Table 1). Cultured bacterial species with the greatest prevalence were *Bacillus licheniformis*, *Corynebacterium xerosis*, and *Staphylococcus epidermidis* (cultured from 7 out of 10 EXT samples). There were 12 bacterial species cultured from the inside of the pregnant tract (Table 2). Four out of 10 cattle (40%) had no bacterial growth from the pregnant tract. For the remaining 6 cattle (60%), there was bacterial growth in at least one location but generally only a single colony was found. The IC_P was the only location without any cultured bacteria (Table 2). Although most (8 out of 12) of the cultured bacterial species identified inside the pregnant tract were also cultured from the EXT (Table 2; Supplementary Table S1), bacteria cultured from the inside of the pregnant uterus were typically not cultured from the EXT from the same animal (Table 2). Bacterial species isolated from either the EXT or inside the pregnancy were known to inhabit skin, soil, gut, mammary gland, and the respiratory and urogenital tracts (Tables 1, 2; Supplementary Table S1).

**Table 1:** List of bacteria species cultured from the external surface of the reproductive tract of 2 or more heifers and cows.

Species	Typical habitat^1^	Number of heifers or cows with isolate		
		Heifers	Cows	Total
*Acinetobacter lwoffii*	Skin	1	1	2
*Aerococcus viridans*	Soil	1	1	2
*Bacillus clausii*	Gut	2	0	2
*Bacillus licheniformis*	Soil	3	4	7
*Bacillus pumilus*	Soil	1	2	3
*Bacillus subtilis*	Soil and gut	2	0	2
*Cellulosimicrobium cellulans*	Soil	1	1	2
*Corynebacterium efficiens*	Soil	2	1	3
*Corynebacterium xerosis*	Skin	5	2	7
*Escherichia coli*	Gut	1	3	4
*Exiguobacterium aurantiacum*	Soil	0	2	2
*Paracoccus* spp.	Soil	2	0	2
*Pseudomonas aeruginosa*	Soil	0	2	2
*Staphylococcus chromogenes*	Mammary	2	1	3
*Staphylococcus devriesii*	Mammary	0	2	2
*Staphylococcus epidermidis*	Skin	3	4	7
*Staphylococcus haemolyticus*	Skin	0	3	3
*Staphylococcus pasteuri*	Skin	1	1	2
*Staphylococcus warneri*	Skin	2	0	2
*Staphylococcus xylosus*	Skin	1	1	2
*Stenotrophomonas maltophila*	Soil	0	2	2
*Streptococcus pluranimalium*	Mammary	2	1	3
*Streptococcus suis*	Gut	1	1	2

^1^Typical habitat were determined using the BacDive database (The Bacterial Diversity Database; https://bacdive.dsmz.de).

**Table 2:** List of bacteria species cultured from the external surface (EXT) or inside the pregnant uterus in heifers and cows, their prevalence in heifers and cows (n, n), and whether the same species was cultured from the external surface (yes or no).

Bacterial genus	Typical habitat^2^	EXT	Prevalence in pregnancy tissue or fluid^1^						
			PLT	IC_P	IC_E	AL_F	AM_F	ABO	MEC
*Bacillus cereus*	Soil	1, 0	0, 0	0, 0	1, 0 (Yes)	0, 0	0, 0	0, 0	0, 0
*Bacillus pumilus*	Soil	1, 2	0, 0	0, 0	0, 0	0, 0	0, 0	0, 0	1, 0 (No)
*Cutibacterium acnes*	Skin	1, 0	0, 0	0, 0	1, 0 (No)	0, 0	0, 0	1, 1 (No, No)	0, 0
*Lactococcus lactis*	Soil	0, 0	1, 0 (No)	0, 0	0, 0	0, 0	0, 0	0, 0	0, 0
*Lysinibacillus fusiformis*	Soil	0, 1	0, 0	0, 0	0, 0	1, 0 (No)	0, 0	0, 0	0, 0
*Macrococcus flavus*	Soil	0, 0	0, 0	0, 0	0, 0	0, 0	1, 0 (No)	0, 0	0, 0
*Staphylococcus epidermidis*	Skin	3, 4	0, 0	0, 0	0, 0	0, 0	0, 0	0, 0	0, 1 (Yes)
*Staphylococcus haemolyticus*	Skin	0, 3	0, 0	0, 0	0, 0	0, 0	0, 0	0, 0	1, 0 (No)
*Staphylococcus hominis*	Skin	0, 0	0, 0	0, 0	0, 0	0, 0	0, 0	0, 1 (No)	0, 0
*Staphylococcus xylosus*	Skin	1, 1	0, 0	0, 0	1, 0 (No)	0, 0	0, 0	0, 0	0, 0
*Streptococcus sanguinis*	Oral cavity	0, 0	0, 0	0, 0	0, 0	0, 0	0, 0	0, 0	0, 1 (No)
*Streptomyces* spp.	Soil	0, 1	0, 1 (No)	0, 0	0, 0	0, 0	0, 0	0, 0	0, 0

^1^The first number is the number of heifers and the second number is the number of cows from which the bacteria were cultured. A “0,1,” for example, indicates that the bacteria was isolated from no heifers and 1 cow at the specific tissue location. The “Yes” or “No” beneath the number is whether the same bacterium was isolated from the external surface of the same heifer or cow. EXT, external surface of the pregnant tract; PLT, placentome; IC_P, intercotyledonary placenta; IC_E, intercaruncular endometrium; AL_F, allantoic fluid; AM_F, amnionic fluid; ABO, fetal abomasum; MEC, meconium. ^2^Typical habitats were determined using the BacDive database (The Bacterial Diversity Database; https://bacdive.dsmz.de).

### 3.2 16S rRNA gene sequencing

#### 3.2.1 Removal of untargeted and contaminant sequences

**Figure 1:**
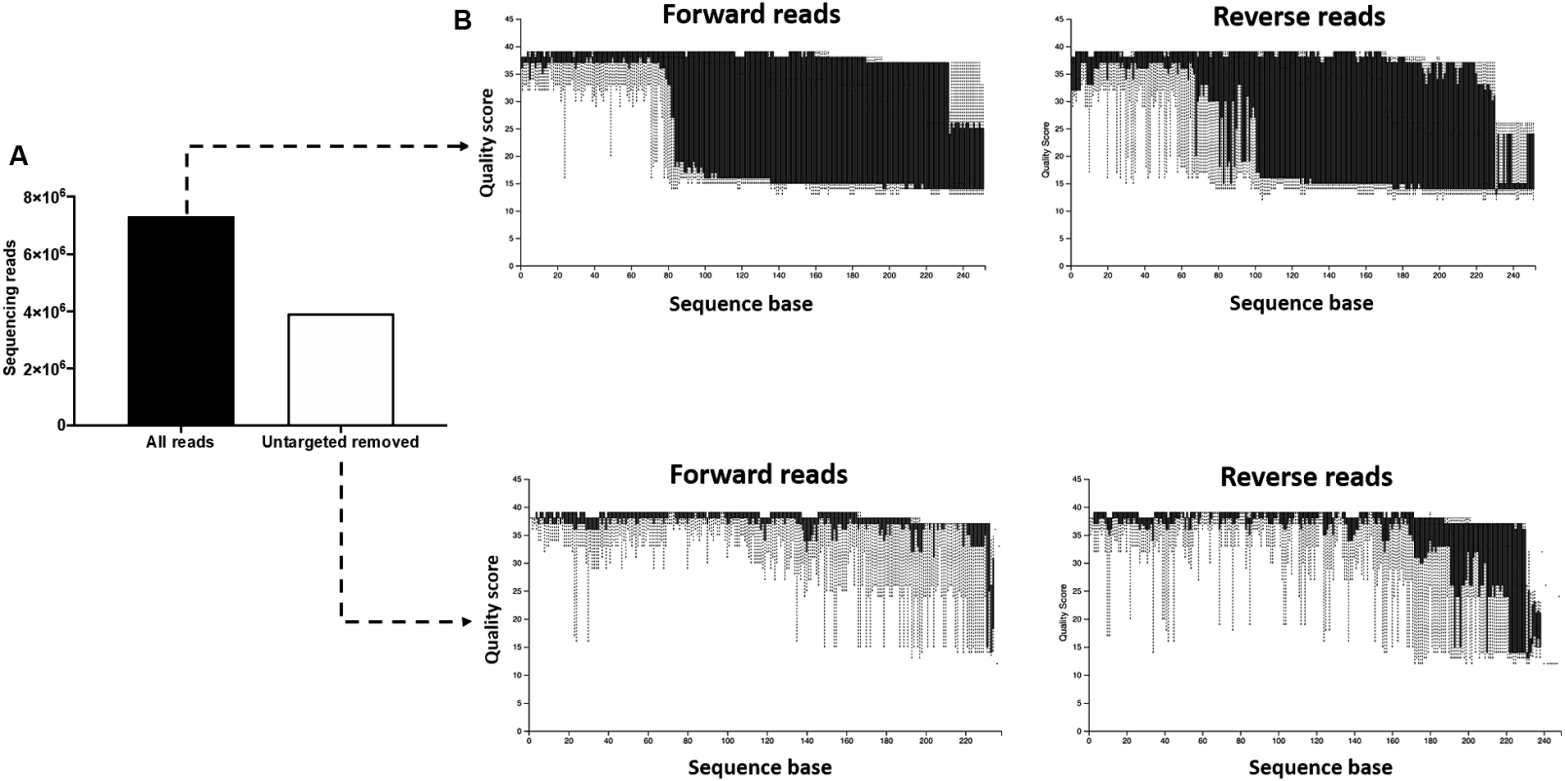
Removal of untargeted sequencing reads. **(A)** The initial library contained 7,324,725 features (sequencing reads). Following removal of sequences not containing the 16S V4 amplification primers (515F/806R), 3,396,929 features were retained for subsequent processing. **(B)** The removal of non-targeted sequences greatly improved the quality scores of the sequences kept for further processing, demonstrating that the non-targeted reads were of low-quality scores.

During the initial processing of the 16S rRNA gene sequencing data, we found that the pregnant bovine uterus had a very low microbial biomass and that a large proportion of the generated library was composed of non-targeted contaminating sequences from the cow and other non-bacterial species. Removing the contaminating sequences [i.e., not containing the 16S V4 amplification primers (515F/806R)] eliminated 3,396,929 sequencing reads [46% of the total initial sequences (7,324,725); Figure 1A] from the subsequent analysis and greatly improved the quality scores of the sequences kept for further processing (Figure 1B). This demonstrated that non-targeted sequences were generally of low-quality scores. Because the amplification primers (515F/806R) can also undesirably bind to non-bacterial DNA, we performed further quality control to eliminate contaminant sequences. In this step, we aligned our sequencing reads to the Green Genes (see Footnote 2) OTU reference sequences (99% sequence identity) and used a threshold of 99% alignment for the query sequence to pass the quality control. Seventy-three contaminant sequences were eliminated in this process (Supplementary Table S2). Sequences filtered out during this step were investigated using the NCBI blast nucleotide database. Predicted matching sequences included regions in bovine, fungal, and viral genomes among others. Importantly, reads from the 73 eliminated sequences accounted for 91.7% of the total reads available prior to quality control. Reads from the targeted bacterial sequences constituted only 8.3% of the total reads after the initial filtering (which removed sequences not containing the amplification primers) and only 4% of the reads from the initial library.

#### 3.2.2 Effect of sample location

**Figure 2:**
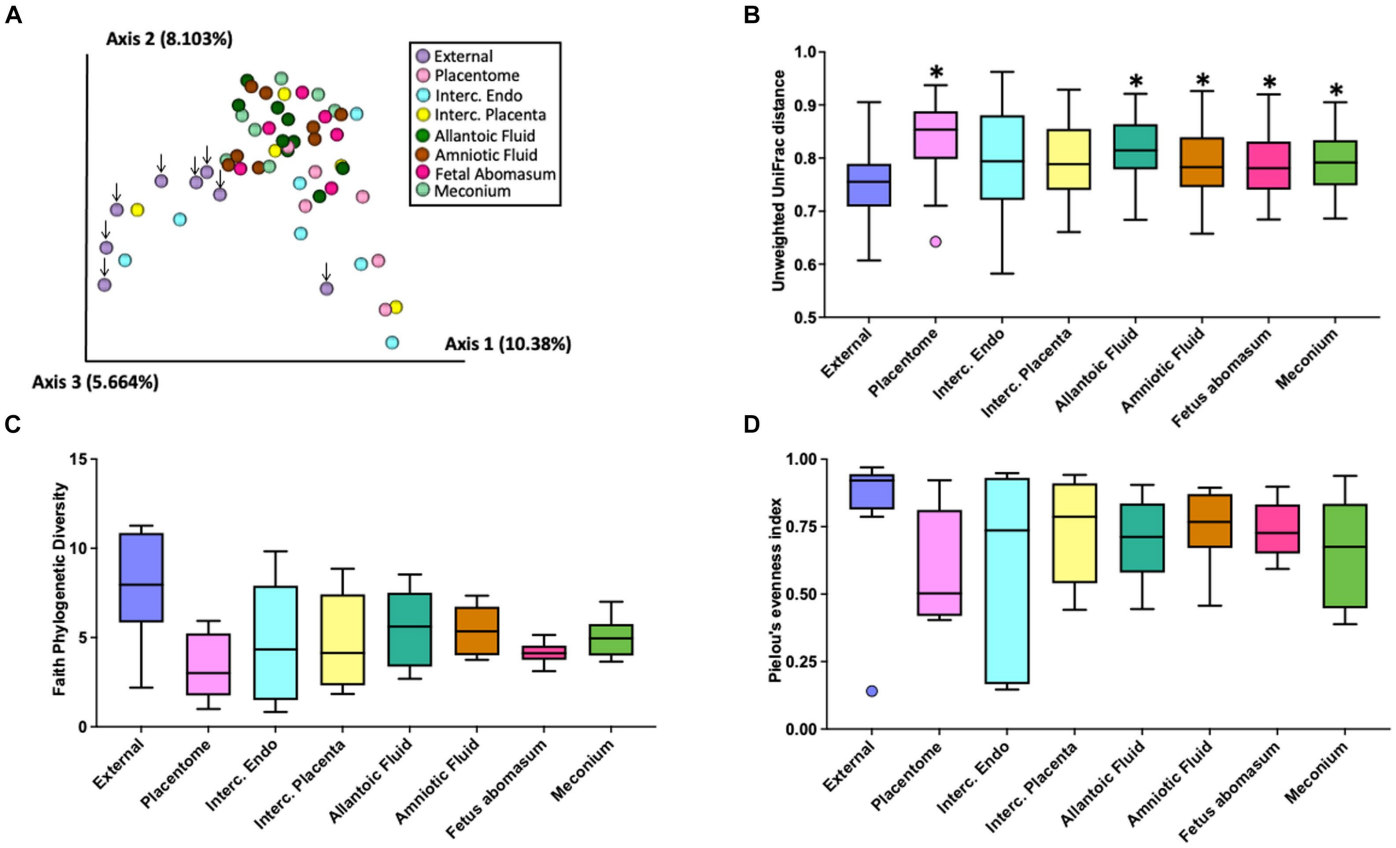
Graphic representation of beta diversity. **(A)** Principal coordinates analysis (PCoA) plot of the unweighted Unifrac distance. Down arrows indicate the location of samples from the external surface. **(B)** Boxplot of the unweighted UniFrac distances for samples collected from the external surface or inside the pregnant uterus. Unweighted UniFrac distance was lower (*q*-value < 0.01) in external (EXT) samples than samples from the allantoic fluid (AL_F), amniotic fluid (AM_F), fetal abomasum (ABO), meconium (MEC), and placentome (PLT). Furthermore, differences in beta diversity (not highlighted in the graph) were also observed in the pairwise PERMANOVA comparisons between samples from AL_F vs. IC_E (*q*-value = 0.04), AL_F vs. PLT (*q*-value = 0.04), AM_F vs. PLT (*q*-value = 0.02), IC_E vs. MEC (*q*-value = 0.01), and MEC vs. PLT (*q*-value = 0.02). Alpha diversity (diversity within samples collected) was evaluated by Faith’s Phylogenetic Diversity **(C)** and Pielou’s Evenness Index **(D)**. Following the Benjamini & Hochberg correction for multiple comparisons [e.g., false discovery rate (FDR)], there was no effect of sample location on Faith’s Phylogenetic Diversity (*q*-value ≥ 0.26) or Pielou’s Evenness Index (*q*-value ≥0.37).

There was a total of 1,133 ASVs and 297,645 16S rRNA gene sequence reads detected across all tissue samples collected from heifers and cows (Supplementary Table S3). The EXT, PLT, IC_P, IC_E, AL_F AM_F, ABO, and MEC were represented by a total of 449, 136, 185, 207, 535, 361, 138, and 236 ASVs and a total of 73,274, 13,148, 6,113, 36,141, 68,689, 77,864, 6,308, and 16,108 sequence reads, respectively. Beta diversity (diversity between samples collected) for heifers and cows together was assessed by PCoA (Figure 2A) based on the unweighted UniFrac distances (considers presence/absence and incorporates phylogenetic distances between the observed organisms). Our positive control samples (EXT; purple dots) clustered separately from samples collected from the inside of the pregnant tract (Figure 2A). The unweighted UniFrac Distance was lower (*q-value* ≤ 0.01) in EXT samples compared with samples from the PLT, AL_F, AM_F, ABO, and MEC (Figure 2B). Differences in unweighted UniFrac distances were also observed among pairwise PERMANOVA comparisons between samples from AL_F vs. IC_E (*q*-value = 0.04), AL_F vs. PLA (*q*-value = 0.04), AM_F vs. PLA (*q*-value = 0.02), IC_E vs. MEC (*q*-value = 0.01), and MEC vs. PLA (*q*-value = 0.02). Alpha diversity (diversity within samples collected) was evaluated by Faith’s Phylogenetic Diversity (Figure 2C) and Pielou’s Evenness Index (Figure 2D). Following the Benjamini & Hochberg correction for multiple comparisons [e.g., false discovery rate (FDR)], there was no effect of sample location on Faith’s Phylogenetic Diversity (*q*-value ≥ 0.26) or Pielou’s Evenness Index (*q*-value ≥ 0.37). According to the differential abundance analysis performed using ANCOM (Mandal et al., 2015), there were no differently abundant ASVs in all pairwise comparisons tested for the distinct sample locations.

#### 3.2.3 Prevalence of individual genera across tissue locations

The 1,133 detected ASV were collapsed to the genus level (the reads from species sharing the same genus were summed together) for further analyses. There were a total of 546 genera present (all ASV) with 210 genera meeting the inclusion requirement used for further analyses [found in at least 2 individuals within at least one tissue and having a minimum of 10 reads across all tissues]. Individual bacterial genera identified by 16S rRNA gene sequencing were defined as “abundant” if greater than 6 cattle (out of 10 total) had sequencing reads within the identified genus. There were 21 (14.8%), 2 (3.7%), 6 (7.6%), 5 (5.1%), 12 (7.8%), 15 (12.1%), 6 (8.3%), and 8 (8.2%) abundant genera for EXT, PLT, IC_P, IC_E, AL_F AM_F, ABO, and MEC, respectively (Supplementary Table S4). Conversely, the number of genera represented in 1 or 2 individuals within a tissue (“rare” genera) were 72 (50.7%), 44 (81.5%), 66 (83.5%), 79 (79.8%), 95 (61.7%), 64 (51.6%), 56 (77.8%), and 63 (64.9%) for EXT, PLT, IC_P, IC_E, AL_F AM_F, ABO, and MEC, respectively.

There were 69 genera represented by at least 10 or more individuals in any tissue. We used the method described by Davis et al. (2018) in an attempt to correct for background reagent contamination in our samples. This method is based on the observation that DNA amplification of 16S rRNA from contaminating species is expected to decrease in a log-linear manner with the total number of sequence reads. We detected 24 genera that were either likely (*p <* 0.05) or potential (0.05 < *p <* 0.10) contaminants using this method (Supplementary Table S5; Supplementary Figure S1). The contaminating genera were removed from the summary of the prevalence of the genus found within the EXT and pregnant uterus (Table 3; Supplementary Table S6).

**Table 3:** List of bacterial genera identified by using 16S rRNA gene sequencing of the inside or external surface (EXT) of the pregnant uterus in heifers and cows, their typical habitat, and their prevalence for 10 cattle total.

Bacterial genus	Typical habitat^2^	Prevalence in pregnancy tissue or fluid^1^							
		EXT	PLT	IC_P	IC_E	AL_F	AM_F	ABO	MEC
*Cutibacterium*	Skin	10	8	8	9	10	10	10	10
*Staphylococcus*	Skin	7	10	7	7	9	9	8	8
*Streptococcus*	Widespread	6	5	6	6	7	8	6	6
*Corynebacterium*	Soil	6	2	6	5	9	7	4	8
*Lactobacillus*	Widespread (Including vagina)	5	5	3	4	5	7	6	6
*Bacteroides*	Gut	7	2	6	6	5	5	2	3
*Anaerococcus*	Widespread (Including vagina)	3	3	1	3	7	6	3	2
*Alistipes*	Gut	9	2	2	2	3	4	0	2
*Lawsonella*	Soil	4	2	2	0	4	4	3	4
*Peptoniphilus*	Vagina	2	0	3	3	7	5	2	1
*WCHB1-41*	Gut	7	0	1	3	5	4	1	2
*Alloprevotella*	Oral cavity	6	0	2	1	2	5	1	5
*Stenotrophomonas*	Soil	2	2	1	2	3	6	2	4
*Actinomyces*	Widespread (soil, skin, vagina)	0	1	1	1	7	4	3	4
*Finegoldia*	Skin and mucous membranes	1	0	1	1	5	5	4	4
*Oscillospiraceae_UCG-005*	Gut	8	0	2	4	1	3	2	1
*Christensenellaceae_R-7_group*	Gut	7	1	1	3	2	3	0	1
*Haemophilus*	Upper respiratory tract	1	0	0	0	3	4	5	5
*Mycoplasma*	Upper respiratory, urogenital	5	3	2	5	0	2	1	0
*Rothia*	Oral cavity, upper respiratory tract	0	0	2	2	3	4	2	5
*Rikenellaceae_RC9_gut_group*	Gut	6	0	2	3	3	2	0	1
*Gemella*	Oral cavity, upper respiratory tract	1	1	1	1	3	3	3	3
*Veillonella*	Oral cavity, gut, urogenital tract	1	1	1	0	5	3	1	4
*Granulicatella*	Oral cavity, gut, urogenital tract	0	1	1	0	2	4	3	4
*Massilia*	Soil	2	1	0	0	6	3	2	1
*Romboutsia*	Gut	5	1	1	2	2	2	1	1
*Lachnospiraceae_NK4A136_gp*	Gut	2	1	1	0	3	3	2	2
*Prevotellaceae_UCG-003*	Gut and vagina	4	0	1	2	3	2	1	1
*Brachybacterium*	Soil	1	0	2	3	3	4	0	0
*Clostridia_UCG-014*	Soil	4	0	1	1	1	4	1	1
*Clostridia_vadinBB60_group*	Soil	5	0	0	1	3	1	0	3
*Clostridium_sensu_stricto_1*	Soil	5	0	1	2	2	1	2	0
*Faecalibacterium*	Gut	1	0	1	0	3	4	1	3
*Lactococcus*	Soil	0	0	1	1	2	6	1	1
*Lachnospiraceae_A2*	Gut	1	0	1	0	4	2	0	3
*Bifidobacterium*	Oral cavity and gut	2	0	1	1	3	2	0	2
*Dietzia*	Soil	3	1	2	1	1	3	0	0
*Fenollaria*	Soil	0	0	1	1	2	3	0	4
*Salinicoccus*	Soil	0	2	2	0	3	1	1	2
*Agathobacter*	Gut	0	0	0	1	4	2	0	3
*Akkermansia*	Gut	4	0	1	1	3	0	0	1
*Bacteroidales_RF16_group*	Gut	6	0	0	2	0	1	0	1
*Ezakiella*	Gut	1	0	0	0	3	3	0	3

^1^EXT, external surface of the pregnant tract; PLT, placentome; IC_P, inter-cotyledonary placenta; IC_E, inter-caruncular endometrium; AL_F, allantoic fluid; AM_F, amnionic fluid; ABO, fetal abomasum; MEC, meconium. There were 10 cattle total and only genera that were represented in at least 10 samples are shown. A complete list is provided in Supplementary Table S6. ^2^Typical habitats were determined using the BacDive database (The Bacterial Diversity Database; https://bacdive.dsmz.de).

The genera that had the greatest prevalence across all sample types including EXT were *Cutibacterium, Staphylococcus, Streptococcus, Corynebacterium* and *Lactobacillus* (Table 3; Supplementary Table S6). There were genera that were prevalent on the external surface but either not detected or had very low prevalence within the pregnancy (e.g., *Lachnospiraceae_UCG-010, Bacteroidales_RF16_group,* and *Luteimonas*) and also genera that were either not detected or with low prevalence on the external surface but found with relatively high prevalence within the pregnancy (e.g., *Peptoniphilus, Actinomyces, Finegoldia,* and *Haemophilus*).

#### 3.2.4 Typical habitats of sequenced genera

For those genera found in two or more EXT samples, the two most common habitats were gut (39% of the sequenced genera) and soil (29% of the sequenced genera). For those genera that averaged a prevalence of >2 across all pregnancy samples, approximately one-half had a typical habitat of oral cavity, respiratory tract, urogenital tract or vagina with a total of 30% identified as either a gut or soil habitat (Table 3).

## 4 Discussion

This study had the specific objective to test for viable bacteria in the pregnant uterus of Holstein cattle and compare these data with data for 16S rRNA gene sequencing. Bacterial culture of the EXT of the pregnant tract resulted in culture of viable bacteria in all animals (100% prevalence) with 87 unique species (Supplementary Table S1). Twenty-three of the species were cultured from more than one animal (Table 1). The typical habitat for the cultured species was soil (environment), skin, mammary gland, and the gut. These bacteria likely contaminated the tract when it was removed from the abdomen of the cow. To our knowledge, this is the only microbiome study of the bovine uterus that specifically examined contamination of the outside of the reproductive tract (EXT) to better understand a potential source of bacterial contamination during tissue collection.

The EXT sample was included because we were concerned that bacteria on the outside of the tract would contaminate the inside of the tract during sampling. We did not find, however, that bacteria on the EXT (Table 1) were readily transferred to the interior of the tract during sampling. We cultured very few bacteria inside the pregnant uterus regardless of sample location (Table 2) and bacteria cultured from the inside of the tract were generally not cultured from the EXT of the same animal. The bacteria that we cultured from the inside of the tract, however, were cultured from the EXT of at least one different animal in the study. There were four species, *Lactococcus lactis*, *Macrococcus flavus*, *Staphylococcus hominis*, and *Streptococcus sanguinis,* that were cultured from the inside of the pregnant uterus but not on EXT samples of any animal. Regarding the existence of a specific microbiome signature in different tissues within the pregnant uterus, there was no consistent pattern of abundance or occurrence (presence/absence) for the bacterial species cultured considering samples from all tissues analyzed in the present study (Table 2). We included components of the placenta (PLT and IC_P), uterus (IC_E), placental fluids (AL_F and AM_F), and fetal locations that were potential reservoirs for bacterial species (ABO and MEC). The overall conclusion was that there are a small number of viable bacteria within the pregnant uterus. This conclusion is similar to that made by several other studies that attempted to culture viable bacteria from pregnant uterus of cow or other species (Guzman et al., 2020; Theis et al., 2020; Husso et al., 2021). Some of the viable species that we identified within the pregnancy (Table 2) have been cultured previously from either the nonpregnant diseased uterus or the pregnant uterus itself (Lyons et al., 2009; Wagener et al., 2015). The bovine uterus evolved with the capacity to sense and eliminate infection (Sheldon et al., 2014). It should perhaps not be surprising, therefore, that a small number of bacteria are present. The more important question is whether these bacteria are present in adequate numbers to have a significant impact on uterine biology as they clearly do in other sites where there are a large number of bacteria (intestine, skin, vagina, etc.). It seems unlikely that such a small number of viable bacteria could have a large impact on the biology of the bovine uterus at mid-pregnancy. This is not to say the bacteria are never involved in the pregnancy as clearly there are species that can infect the uterus early postpartum (Sheldon et al., 2020) and cause abortion in pregnant animals later postpartum (Kirkbride, 1993). A large commensal role for the pregnancy microbiome in the bovine, nonetheless, seems unlikely.

The strength of classical bacteriology is that only living organisms are identified. A weakness of bacterial culture is that most bacteria fail to grow when standard culture techniques are applied (Amann et al., 1995). Moreover, certain microorganisms thrive under artificial culture conditions thereby inhibiting the growth of others and potentially resulting in an underestimation of the overall community diversity (Byrd et al., 2018).

The purpose of the 16S rRNA gene sequencing was to identify the entire microbiome which includes cultivable and noncultivable species. Despite efforts to minimize contamination during sample collection and processing, pregnancy tissues have an inherited low microbial biomass, and therefore contamination during sample collection, DNA extraction, and library preparation and sequencing likely introduced spurious taxa not present in the original samples. Despite of these limitations, and as expected, the 16S rRNA gene sequencing returned ASVs across all sampled sites. The number of ASVs was greatest for EXT and AL_F. The PLT and ABO had the least number of ASVs. The total number of sequence reads was highly variable between animals and similar across all sites. The EXT samples clustered separately from the remaining samples which were collected from the inside of the pregnant uterus in the PCoA plot (Figure 2A). Additionally, EXT samples were less similar to one another (Figure 2B) when compared with the tissue collected from the pregnancy itself. These results are consistent with the conclusion of other papers that found a diverse low biomass microbiome within the uterus (Baker et al., 2018). The reduced similarity on the external surface (Figure 2B) likely reflects the unique contamination patterns for cows slaughtered on different days. Despite apparent differences in diversity at the population level (Figure 1A), differential abundance analysis at the ASV level using ANCOM failed to detect any statistical difference between the microbial populations at different sites. This demonstrates the lack of a robust microbiome signature in each tissue investigated. Even though our study was not designed to detect taxa affecting reproductive health, our *in vitro* culture and metataxonomic analyses yielded no evidence of bacteria associated with reproductive tract infection in dairy cattle, as we failed to detect increases in abundance of pathogenic bacteria generally associated with uterine infection (e.g., *Fusobacterium necrophorum*, *Bacteroides* spp., *Porphyromonas*, *Escherichia coli*, *Trueperella pyogenes*) (Galvão et al., 2019; Sheldon et al., 2020; Moraes et al., 2021).

The number of sequence reads was low (median of 652 reads per sample) as has been reported for many other studies of the pregnant or non-pregnant uterus of the cow and other species (Lietaer et al., 2021). For comparison, similar work on d 7 postpartum uterus (including metritic and non-metritic cows) returned a median of 220,548 reads per sample and later postpartum (d 30) the median was 9,343 reads per sample (Moraes et al., 2024). Microbiome studies of tissues with a low microbial biomass are difficult to conduct because the potential for sample contamination from laboratory reagents is high (Eisenhofer et al., 2019; O’Callaghan et al., 2020). This has led to numerous recommendations with respect to controlling and correcting for contaminants. In our case, we were primarily concerned that we would contaminate the tissue samples during sample collection and did not initially include a true negative control (blank tube or similar) at the time of collection. We identified contaminating genera using the statistical technique described by Davis et al. (2018) (Supplementary Table S5; Supplementary Figure S1) and removed 24 contaminating genera from our summary tables (Table 3; Supplementary Table S6). Although there were some bacterial genera that were present across all locations with a moderate (30 to 50%) or abundant (>50%) prevalence (Table 3) this was generally not the case. Specific genera may be abundant on the EXT (positive control sample) and rare or absent inside the pregnant tract. Conversely, specific genera may be rare or absent on the outside of the tract but unique to specific locations inside the pregnancy (Table 3). Our data bear some similarity to a recent publication on amnionic fluid and meconium from a similar stage of bovine pregnancy (Husso et al., 2021). At the genus level, *Cutibacterium, Staphylococcus, Alloprevotella Corynebacterium, Streptococcus,* and *Lactobacillus* were highly prevalent in the AM_F and MEC from both studies. We also noted that the genera *Cutibacterium, Staphylococcus* and *Streptococcus* that were prevalent in our 16S rRNA gene sequence from the pregnancy (Table 3) were representative of 6 of 12 of the viable bacteria that we cultured (Table 2). *Cutibacterium acnes* was the only bacterium that we cultured from more than one pregnancy (one heifer and one cow ABO) and was also the most prevalent of all of the 16S rRNA genera. *Cutibacterium acnes* (formerly *Propionibacterium acnes*) has been found by others to be abundant in 16S rRNA sequence and present in bacterial culture of AM_F (Stinson et al., 2019). It has been reported to cause abortion in cattle (Lyons et al., 2009).

Many of the genera that were found on the EXT by using 16S rRNA gene sequencing had a typical habitat of gut and soil. The typical habitat for the pregnancy-related tissues was either oral cavity, respiratory tract, urogenital tract or vagina (Table 3). A link between the microbiome of the oral cavity and the pregnancy was reported in one of the first reports of a microbiome of pregnancy (Aagaard et al., 2014). Their conclusion was that the placental microbiome is likely established by hematogenous spread of oral microbiome during pregnancy. We did not test the specific hypothesis that the oral microbiome can be transferred to the pregnancy in our study. The recent demonstration that fluorescently-labeled bacteria that are administered orally, intravenously, or vaginally will populate the ovine fetus during late pregnancy (Yu et al., 2021) clearly demonstrates that exposure of the dam to bacteria through an oral or other route can affect the microbiome of the pregnancy.

Although cultured bacteria were typical inhabitants of the environment (soil) and skin (Supplementary Table S1; Table 1), we noted that the 16S rRNA genera that we detected were more typical inhabitants of the gastrointestinal tract; particularly when the EXT was tested. Whether this reflects the suitability of culture conditions for these specific genera or the possibility that bacterial DNA translocation from the gut to the EXT is occurring should be explored further. Others have hinted at the possibility that gut bacteria may be transmitted to the uterus via the bloodstream (Jeon et al., 2017), although in our study these bacteria did not appear to be viable (failed to culture). The possibility that the bacterial DNA (non-viable) from the intestine contaminated the abdomen and external surfaces of organs should be explored further.

## 5 Conclusion

We conclude that there are a small number of viable bacteria in the pregnant uterus. The metataxonomic (16S rRNA gene sequencing) analyses indicated a low microbial biomass within the pregnant uterus. These results are consistent with recent studies of the pregnant bovine uterus that demonstrate the presence of a microbiome but leave open the question of whether there is adequate microbial mass to significantly affect the biology of the normal healthy bovine pregnancy.

## Data availability statement

The sequence files and associated metadata for all samples utilized in this study have been securely deposited in the NCBI Sequence Read Archive (SRA) repository (BioProject accession: PRJNA1097682).

## Ethics statement

The animal study was approved by Study procedures were approved by the University of Missouri Institutional Animal Care and Use Committee (Protocol number 9635). The study was conducted in accordance with the local legislation and institutional requirements.

## Author contributions

JM: Investigation, Methodology, Formal analysis, Writing – original draft, Writing – review & editing. TG: Investigation, Methodology, Supervision, Writing – review & editing. AE: Investigation, Methodology, Supervision, Writing – review & editing. SP: Investigation, Project administration, Supervision, Writing – review & editing. MC: Investigation, Writing – review & editing. ML: Conceptualization, Formal analysis, Funding acquisition, Investigation, Methodology, Project administration, Supervision, Writing – original draft, Writing – review & editing.

## Footnotes

^1^ https://qiime2.org

^2^ https://greengenes.secondgenome.com

^3^ https://blast.ncbi.nlm.nih.gov/Blast.cgi

^4^ https://bacdive.dsmz.de
